# Unique subgingival microbiota associated with periodontitis in cirrhosis patients

**DOI:** 10.1038/s41598-018-28905-w

**Published:** 2018-07-16

**Authors:** Anders Jensen, Lea Ladegaard Grønkjær, Palle Holmstrup, Hendrik Vilstrup, Mogens Kilian

**Affiliations:** 1 Department of Biomedicine, Faculty of Health, Aarhus University, Aarhus, Denmark; 2 Department of Hepatology and Gastroenterology, Aarhus University Hospital, Aarhus, Denmark; 3 Section of Periodontology, Microbiology, and Community Dentistry, Department of Odontology, Faculty of Health and Medical Sciences, University of Copenhagen, Copenhagen, Denmark

## Abstract

Liver cirrhosis is a severe disease with major impact on the overall health of the patient including poor oral health. Lately, there has been increasing focus on oral diseases as cirrhosis-related complications due to the potential impact on systemic health and ultimately mortality. Periodontitis is one of the most common oral diseases in cirrhosis patients. However, no studies have investigated the composition of the subgingival microbiome in patients suffering from periodontitis and liver cirrhosis. We analysed the subgingival microbiome in 21 patients with periodontitis and cirrhosis using long-reads Illumina sequencing. The subgingival microbiota was dominated by bacteria belonging to the *Firmicutes* phylum and to a lesser extend the *Actinobacteria* and *Bacteroidetes* phyla. Bacteria usually considered periodontal pathogens, like *Porhyromonas ginigivalis*, *Tannerella forsythia*, *Treponema denticola*, generally showed low abundancy. Comparing the microbiota in our patients with that of periodontitis patients and healthy controls of three other studies revealed that the periodontitis-associated subgingival microbiota in cirrhosis patients is composed of a unique microbiota of bacteria not normally associated with periodontitis. We hypothesise that periodontitis in cirrhosis patients is a consequence of dysbiosis due to a compromised immune system that renders commensal bacteria pathogenic.

## Introduction

Periodontitis is a bacterially induced inflammatory disease that affects the supporting tissues of teeth, characterized by loss of connective tissue and bone with ensuing deepening of the periodontal pockets. Untreated, periodontitis may result in loosening of teeth with discomfort, impaired mastication, pain, and ultimately tooth loss^1,2^. Periodontitis in adults is caused by loss of the natural balance between the oral microbiota and host resistance (dysbiosis) due to life style factors, e.g. insufficient oral hygiene and tobacco smoking, combined with genetically and disease-associated jeopardization of host defence. Ensuing changes in the relative abundances of members of the complex microbiota that accumulate on the teeth toward pro-inflammatory species trigger a host response that includes release of inflammatory exudates, which further stimulate changes in the microbiota. In some cases, this dysbiosis leads to a situation where a destructive inflammatory response results in degradation of connective tissue and periodontal bone^3–5^. In addition, environmental and genetic factors secondary to these inflammatory and destructive events may continue to support a pathogenic microbiota and perpetuate the cycle of events^4^.

Poor oral health, including oral diseases such as periodontitis, is prevalent in patients with liver cirrhosis^6–8^, which is the final pathway for several liver diseases defined as the histological development of regeneration nodules surrounded by fibrous tissue^9^. The prevalence of oral diseases is not a trivial problem as it may have systemic implications. Thus, studies have assessed the association between periodontitis and several systemic diseases and an increasing number of studies have suggested that oral diseases may affect the course of systemic diseases^10^. Thus, our previous cross-sectional studies in patients with cirrhosis indicated an association between oral diseases and a higher nutritional risk score, systemic inflammation activation, increased frequency of cirrhosis-related complications and even higher mortality in cirrhosis patients suffering from periodontitis^11–13^. Possible mechanisms of the systemic effects include spreading of oral bacteria and bacterial products, either by aspiration or through the bloodstream facilitated by periodontal inflammation^14^. Moreover, a spill over of inflammatory cytokines may enter the circulation and induce and perpetuate systemic effects^15^.

In recent years, numerous studies have shown that the human microbiome is involved in health and diseases in humans and dysbiosis of the human microbiome has been associated with several diseases like obesity, diabetes and neurological disorders^16–20^. Also, oral diseases like periodontitis have been associated with a dysbiosis of the oral microbiome^5^. Several studies have shown that liver cirrhosis may cause dysbiosis of the gut microbiome and changes associated with the severity and outcome of the liver cirrhosis^21–24^. Some studies have shown that the dysbiosis of the gut microbiome in liver cirrhosis patients is mostly caused by increased abundancy of oral bacteria compared to healthy controls^24–26^. Only a few studies, however, have examined the oral microbiome in patients with cirrhosis^27,28^. Bajaj *et al*.^27^ found dysbiosis of the salivary microbiome in patients with cirrhosis with hepatic encephalopathy, which may be caused by mucosal immune interface changes. However, no studies have examined the subgingival plaque microbiome in patients with alcohol-induced liver cirrhosis and periodontitis.

To have a more thorough understanding of the complex role of the oral hygiene and especially periodontitis in the outcome of liver cirrhosis patients that we have seen in our companion studies, it is also of great importance to have a comprehensive mapping of the subgingival microbiome from patients with periodontitis and liver cirrhosis.

The aim of the study was therefore to make a comprehensive mapping of the subgingival plaque microbiome in liver cirrhosis patients with periodontitis. Our results show that the subgingival microbiota in liver cirrhosis patients with periodontitis is unique and that bacteria normally associated with periodontitis are found only in very low proportions. We hypothesise that this may be due to an altered immune status in liver cirrhosis patients allowing bacteria not normally associated with periodontitis in otherwise healthy patients to induce periodontitis.

## Methods

This clinical part of this study was conducted between May and August 2015 at Aarhus University Hospital, Denmark. Eligible Patients were consecutively enrolled at the Department of Hepatology and Gastroenterology for a program on oral health and oral infections in patients with cirrhosis^11,12^. The present study focused on the oral microbiota.

Eligible patients were adult men (N = 16) and woman (N = 5) with an established diagnosis of cirrhosis regardless of aetiology and severity. The diagnosis of cirrhosis was based on medical history, physical examination, liver biopsy and/or pertinent clinical-biochemical, and ultrasonographic findings.

### Oral Examination

At study entry, a standardized oral examination was performed and combined with the collection of subgingival plaque from two pathologic periodontal pockets (see below) in two different quadrants. Clinical probing depths (PD) of the gingival pockets were measured parallel to the longitudinal axis of the tooth from the free gingival margin to the tip of the periodontal probe. Clinical attachment level (CAL) of the gingiva was defined as the distance from the cemento-enamel junction (CEJ) to the tip of the periodontal probe. PD and CAL were registered at six sites per tooth, measured in mm, and recorded to the nearest mm. All samples were taken from sites deeper than 4 mm and up to 6 mm. Bleeding on probing (BOP) was registered if bleeding occurred within 15 seconds after probing. Periodontitis was defined and classified as mild, moderate or severe, as described by the working group of the Centre for Disease Control and Prevention in collaboration with the American Academy of Periodontology (CDC-AAP)^29^. For a complete overview of the clinical characteristics of the cirrhosis patients see Tables 1 and S1.

**Table 1:** Characteristics of the patient cohort (n = 21).

Age, years (median, IQR)	62 (59–74)
Female/Male	5/16
Cirrhosis etiology	
Alcohol	15
Cryptogenic	4
Autoimmune or cholestastic	2
Cirrhosis severity (median, IQR)	
Child-Pugh score	9 (7–11)
Model of End-Stage Liver Disease score	13 (8–20)
Smoker status	
Current/Former/Never	10/7/4
Daily alcohol use	
Yes/No	10/11
Charlson comorbidity index	
0	12
1	7
2	1
3+	1
Medical use at examination	
Lactulose	10
Pantoprazole	7
Rifaximin	4
Antibiotics use within the last ½ year	12
Periodontal measures (median, IQR)	
Number of teeth	24 (16–27)
Probing depth all teeth, mm	3.11 (2.72–3.85)
Clinical attachment level all teeth, mm	3.43 (2.83–4.25)
Bleeding on probing, all sites (%)	50 (14–92)
Periodontitis	
CDC-APP mild cases	2
CDC-APP moderate cases	8
CDC-APP severe cases	11

IQR, interquartile range.

After the oral examination, the sites to be sampled were selected. The plaque collection procedure was preceded by removal of supragingival plaque and the gingival margin was wiped dry with a sterile cotton pellet. Subsequently, a curette was inserted into a site of the gingival pocket with a depth of 4 mm or more, and the plaque sample was removed by a single curette stroke. The procedure was repeated for another gingival pocket in a different quadrant. The two plaque samples from each patient were pooled in 750 µl PowerLyzer™ PowerSoil® Bead Solution buffer and stored at −80 °C. An authorised dental hygienist undertook the oral examinations and plaque collections. Prior to study start, she was trained by an experienced clinical examiner in periodontitis from the Department of Odontology, Aarhus University, Denmark.

### DNA extraction

At arrival of the samples to the laboratory at Aarhus University, DNA was immediately extracted from the samples using PowerLyzer PowerSoil DNA Isolation Kit (MO-BIO, Carlsbad, CA) and according to the manufacturer’s instructions with the exception that a Fastprep FP120 (Qbiogene, Carlsbad, CA) was used to release DNA. Briefly, after thawing, 60 µl of the C1-solution were added to the sample and the samples were then transferred to a Powerlyser Glass Bead Tube. DNA was released from the cells using a Fastprep FP120 machine (Qbiogene, Carlsbad, CA) at 5.5 ms^−1^ for 30 seconds. The treatment was repeated three times and the samples were cooled in ice between the treatments. After the bead beating step the user manual was followed from paragraph number 7. DNA was eluted in 100 µl C6 buffer supplied from the kit. The extracted DNA was then examined for concentration and purity on a NanoDrop 2000 spectrophotometer (Thermo Scientific, Waltham, MA) and all samples fulfilled the requirements of GATC-Biotech of a total DNA concentration of 1–50 ng/µl and an OD 260/230 >1.8; OD 260/230 >1.9.

### PCR amplification and Sequencing

Purified DNA was send to GATC-Biotech (Konstanz, Germany) for PCR amplification and sequencing using their internal protocol. Briefly, the V1-V3 region of the 16S rRNA gene was amplified using the 27F primer (5′-AGAGTTTGATCCTGGCTCAG-3′) and the 534R primer (5′-ATTACCGCGGCTGCTGG-3′) resulting in an amplicon of approximately 508 bp. After initial amplification of the V1–V3 region, the PCR products were purified and the tagged adapters were added through fusion primer PCR. Preparative gel purification was performed before loading the library on the sequencing machine. Due to known problems with the V3 MiSeq chemistry on 2 × 300 bp (see http://blog.Mothur.org/2014/09/11/Why-such-a-large-distance-matrix/ for discussion of the problems), the samples were sequences with an optimized protocol for 2 × 300 bp paired-end sequencing on the Illumina HiSeq. 2500 sequencing system (San Diego, CA, USA). This method has been developed jointly by GACT-Biotech and the Illumina company and has been accredited according to ISO17025. DNA extraction controls as well as positive and negative controls for PCR reactions were included. Because extraction controls showed no PCR products, they were not included for sequencing.

### Data processing and analysis of the cirrhosis patient samples

**Figure 1:**
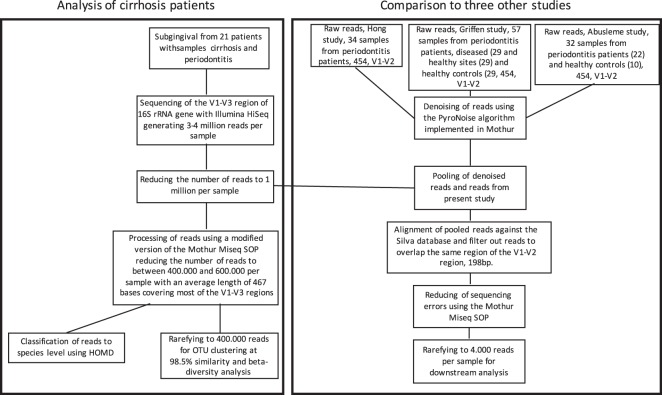
Flowchart summarizing the processing of the reads from the cirrhosis patients and the comparison with reads of downloaded datasets from the Griffen study^35^, the Hong study^37^ and the Abusleme study^36^.

To reduce the error rate of the reads, processing of raw reads from the Illumina platform was done using the Mothur software ver. 1.39.1 and following the MiSeq SOP (https://www.Mothur.org/wiki/MiSeq_SOP)^30^ with some modifications. Initially, the paired-end reads were combined using the command “make.contig” generating 3–4 million reads per sample. Due to the available computer power, the number of reads was then randomly reduced, using the Sub.sample command in Mothur, to exactly 1 million reads per sample similar to the number of reads if the samples were sequenced on the Illumina MiSeq sequencing platform. The combined reads were then trimmed off the primer sequence and sequences containing ambiguous bases or containing longer stretched of homopolymers than eight were discharged from further analysis. The reads were afterwards aligned against the Silva reference alignment ver. 128 and the resulting alignment was filtered so that all of our reads only overlapped the same region. To further reduce the number of sequencing errors, a preclustering step implementing a pseudo-single linkage algorithm originally developed by Huse *et al*.^31^ with the “diff” variable set to four was performed. Chimeras were removed using the build-in version of the UCHIME algorithm in Mothur^32^. Lastly, all singletons, duplicates, and triplicates were removed before final analysis to preclude inclusion of sequences from potential contamination or sequence errors that have not been detected in the previous steps. The resulting reads were then classified with the classify.seqs command using the Bayesian method and the taxonomic outline ver. 14.51 from the Human Oral Microbiome Database (HOMD) (http://www.homd.org/)^33^. The confidence cut-off was set to 80%. HOMD is a curated database of known oral bacterial species, and allows one to classify the sequences to species level. All samples were then rarefied to 400,000 sequences per sample using the sub.sample command in Mothur and clustered into operational taxonomic units (OTU) defined by a 98,5% similarity level using the opticlust clustering method in Mothur. The 98.5% similarity level was chosen as many named oral bacterial species have high sequence identity in their 16 S rRNA genes, particularly among the streptococci, *Actinomyces* and *Neisseria*. Therefore, a number of different species are likely to be combined into the same OTU when applying the more commonly used distance of 0.03, as previously discussed^34^. The resulting OTUs were then classified using the classify.otu command in Mothur and the HOMD database. The workflow is outlined in Fig. 1.

To identify potential clinical and medical confounding factors that may affect the microbial composition in our samples, Principal Coordinates (PCoA) plots based on ThetaYC distances of the OTUs clustered at a 98.5% similarity level generated were visualized in R ver. 3.3.2. The factors tested for confounding effect were: Gender, smoking, intake of alcohol, diagnosis leading to cirrhosis, severity of periodontitis, use of antibiotics within the last six month and lactulose use.

### Comparison of our data set with other periodontitis microbiome data sets

To compare our data with other microbiome data sets from patients with periodontitis and from healthy controls with no periodontitis, we downloaded raw reads from the studies of Griffen *et al*.^35^, Abusleme *et al*.^36^, and Hong *et al*.^37^ from the Sequence Read Archive (SRA) at NCBI (SRA accession numbers SRP009299, SRP012422, and SRP038001, respectively). The following patient groups were included in the comparison: Periodontitis patients, deep sites, periodontitis patients, shallow sites, and healthy controls from the Griffen study, periodontitis patients and healthy controls from the Abusleme study, and periodontitis patients from the Hong study. Because of the differences in amplified 16S rRNA regions and read lengths across studies, all raw sequence reads were reprocessed in Mothur v.1.39.5 using the Schloss standard operating procedure (SOP) for 454 pyrosequencing data (http://www.Mothur.org/wiki/454_SOP)^38^. After the initial denoising of the data sets with the implemented version of the PyroNoise software^39^ in Mothur. All data sets, including our own data, were then pooled and treated collectively. The pooled sequences were first aligned to the Silva reference alignment ver. 128 and the resulting alignment was then filtered so that all of our sequences only overlapped the same region. The reads in the resulting alignment were, on average, 198 bp. covering the V1 and V2 region of the 16S rRNA gene. To further reduce sequencing errors, the preclustering step with the “diff” variable set to two was performed. Chimeras were removed using the UCHIME algorithm. Lastly, all singletons were removed before analysis to preclude inclusion of sequences from potential contamination or sequencing errors that had not been detected in the previous steps. All samples were rarefied to 4000 sequences per sample prior to downstream analyses using the Sub.sample command in Mothur. We choose to rarefy our samples as this method for normalization is still one of the best methods available especially if the library sizes very considerably (>10x on average) between groups as shown by Weiss *et al*.^40^. Four thousand reads were chosen as a compromise between keeping most of the samples and keeping most of the diversity as shown from the rarefaction curves on Fig. S1. Samples that did not fulfil the minimum number of reads were excluded from analysis resulting in 21 cirrhosis samples, 34 periodontitis samples from the Hong study, 25 periodontitis samples, deep sites and from the Griffen study, 26 periodontitis samples, shallow sites, from the Griffen study, 24 healthy control samples from the Griffen study, 18 periodontitis samples from the Abusleme study, and nine healthy control samples from the Abusleme study.

Sequences from all the included data sets including our own data (this time using the 198 bp of the V1–V2 region) were taxonomically classified using the Bayesian method and the taxonomic outline ver. 14.51 from HOMD. The confidence cut-off was set to 80%. Sequences were then clustered into operational taxonomic units (OTU) defined by a 98.5% similarity level using the opticlust clustering method. The resulting OTUs were then classified using the classify.otu command in Mothur and the HOMD database. Shannon diversity index was calculated in Mothur and Student’s t-test was used to detect differences of the Shannon diversity index between the groups. Differences were considered significant at p < 0.05. Linear discriminant analysis (LDA) coupled with effect size measurement (LEfSe) to detect differentially abundant bacterial taxa (phyla, genera, species) between groups. The alpha values for the factorial Kruskal–Wallis and pairwise Wilcoxon tests were set to 0.05 and the LDA score threshold for discriminative features was set to 3.5. The online version of the LEfSe program were used^41^ (https://huttenhower.sph.harvard.edu/galaxy/). Principal Coordinates (PCoA) plots based on Unweighted Unifrac distances generated from a phylogenetic tree produced in the Clearcut program^42^ and on ThetaYC distances of the OTUs clustered at a 98.5% similarity level were vizualised in R ver. 3.3.2. To test if separation of the defined groups visualized by the Principal Coordinates (PCoA) plots was statistically significant, a PERMANOVA (Permutational Multivariate Analysis of Variance) test was performed using the adonis function in the vegan package (version 2.4–4) implemented in R with 999 permutations. Differences were considered significant at p < 0.05. The workflow is outlined in Fig. 1 and clinical and sequence characteristics for all samples of the seven data sets are shown in Tables 2 and S2.

**Table 2:** Clinical and sequence characteristics for all dataset used for comparison.

Group	No. Samples	No. samples used for analysis	Patient and clinical characteristics				Sequence characteristics						
			Age	Women (%)	Probing depth, all teeth (mean, mm)	Probing depth sampled sites (mm)	Sequencing technology	Variable regions sequenced	Variable regions used for analysis	Sequence alignment lenght (bp, average)	Sequences before rarefying (bp, mean)	OTU’s (98.5%, average)	Good’s coverage
Cirrhosis patients	21	21	62 (median)	24	3.2	≥4	Illumina HiSeq	V1–V3	V1–V2	198	678,892	156	0.97
Hong periodontitis	34	34	52 (mean)	35.3	3.2	7	454 FLX	V1–V2	V1–V2	198	6,263	149	0.98
Griffen periodontitis deep sites	29	25	54.1 (mean)	34.5	ND	6.2	454 FLX	V1–V2	V1–V2	198	5,331	182	0.98
Abusleme periodontitis	22	18	42.5 (mean)	54.5	3	5	454 FLX	V1–V2	V1–V2	198	5,839	122	0.99
Griffen periodontitis shallow sites	29	26	51.3 (mean)	34.5	ND	3.2	454 FLX	V1–V2	V1–V2	198	5,371	169	0.98
Griffen healthy controls	29	24	54.1 (mean)	65.5	ND	2.81	454 FLX	V1–V2	V1–V2	198	5,797	115	0.99
Abusleme healthy controls	10	9	34.1 (mean)	80	1.5	ND	454 FLX	V1–V2	V1–V2	198	6,801	64	0.99

### Availability of data and material

All raw reads were deposited to the NCBI Sequence Read Archive (SRA) with the accession number SRP110002.

### Ethics approval and consent to participate

The study was performed according to the Declaration of Helsinki. The Ethical Committee of Central Denmark Region approved the study (journal No. 1-10-72-128-12). Written, informed consent was obtained from all patients included in the study.

## Results

### Cirrhosis patients

**Figure 2:**
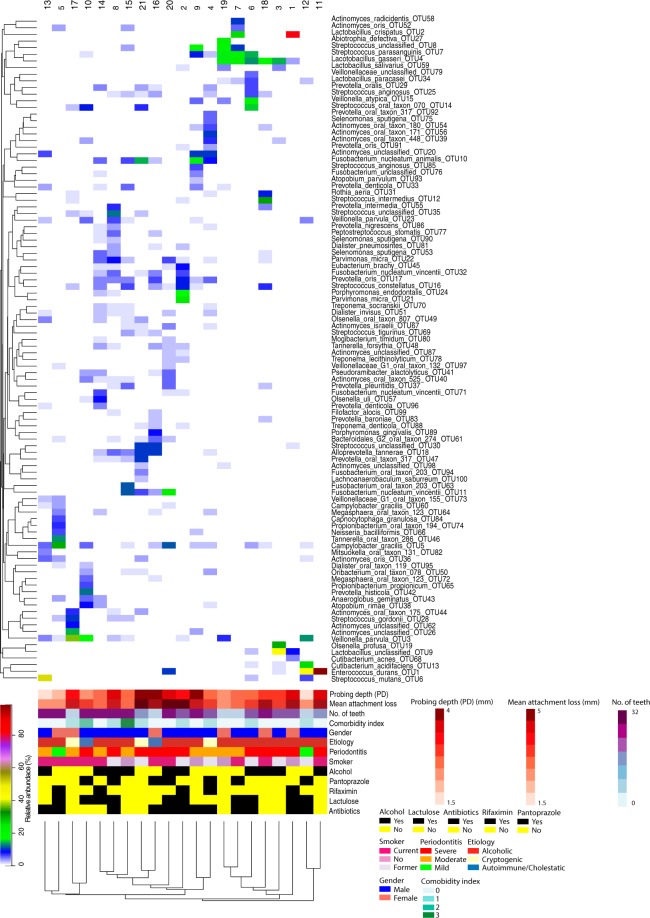
Heatmap of the relative abundances for the top 100 OTUs found in the subgingival microbial communities of the patients with periodontitis and liver cirrhosis. Patients are shown in rows, while OTUs appear in columns. Color scale for the heatmap is shown in the top left with the most abundant OTUs in red and the least abundant in white. Patients are organized according to results of hierarchical clustering (complete linkage) of microbial communities based on OTU relative abundances. Color bars in the left depict the medical and clinical characteristics for each patient. Color scales for metadata appear at the bottom.

Of the initial 1,000,000 sequences in each sample, the final number of sequences was reduced to between 402,937 and 642,319 (mean 577,389) sequences with a mean length of 467 bases after our rigorous sequence processing to reduce the numbers of sequence errors. The number of observed OTUs at a 98.5% sequence similarity ranged from 69 OTUs in sample 6 to 453 OTUs in sample 16 with a mean of 218 OTUs (Table 3). The total number of OTUs observed across all samples was 1653 with only 10 OTUs shared between all samples. The 25 most abundant OTUs accounted for 52% of all the included sequences. The two most dominant OTUs were classified as *Enterococcus durans* and *Lactobacillus crispatus* but both were only found in a few samples. However, *E*. *durans* (OTU1) comprised more than 96% of all sequences of sample 11, while *L*. *crispatus* (OTU2) comprised almost 78% of all the sequences of sample 1 (Table S3). The relatively large variation in the number of OTUs in the samples was also reflected in the Simpson’s inverse diversity index, which ranged from 1.62 in sample 1 to 53.54 in sample 8 and with a mean of 17.29 (Table 3). A heatmap generated from the 100 most abundant OTUs clearly shows that, in most samples, a few OTUs were present in large proportions (>1%) (Fig. 2). In addition, when clustering the samples according to the presence of OTUs, no groupings with any clinical or medical parameters were found (Fig. 2). To further investigate whether any differences in the bacterial community structure between our samples were associated with the clinical or medical characteristics of the included patients, we visualised the community structure using a PcoA plot generated from clustering of the sequences at 98.5% sequence similarity and the ThetaYC calculator (Fig. S2A–F). Although the number of samples in each category is small, the differences in the community structure observed between the included patients were probably not due to differences between subjects in a single parameter alone. However, the diversity of clinical and medical characteristics of the samples may increase the overall variation of the community structure as indicated by the low number of shared OTUs between all samples.

**Table 3:** Sample characteristics after reduction of sequencing errors.

Sample	No. of sequences	Good’s coverage	OTUs (98.5%)	invsimpson diversity index	npshannon diversity index
1	622,881	1.00000	93	1.6	1.0
2	605,126	1.00000	256	11.4	3.2
3	402,937	1.00000	71	3.5	1.7
4	578,223	1.00000	218	27.7	3.8
5	626,181	1.00000	323	14.4	3.7
6	594,296	0.99999	69	8.8	2.5
7	490,496	0.99999	123	6.0	2.1
8	538,865	0.99999	402	53.5	4.6
9	580,848	0.99999	171	15.9	3.3
10	626,790	0.99999	122	14.5	3.0
11	628,812	1.00000	130	1.1	0.3
12	642,319	0.99999	112	3.8	1.7
13	638,673	1.00000	181	4.9	2.7
14	542,274	0.99999	299	48.7	4.3
15	586,535	1.00000	402	36.6	4.3
16	555,276	0.99999	453	48.3	4.6
17	612,666	0.99999	252	7.6	2.9
18	633,027	0.99999	216	7.8	2.9
19	479,118	1.00000	142	8.5	2.6
20	591,593	0.99999	270	14.8	3.4
21	548,228	0.99999	277	23.6	3.7

**Figure 3:**
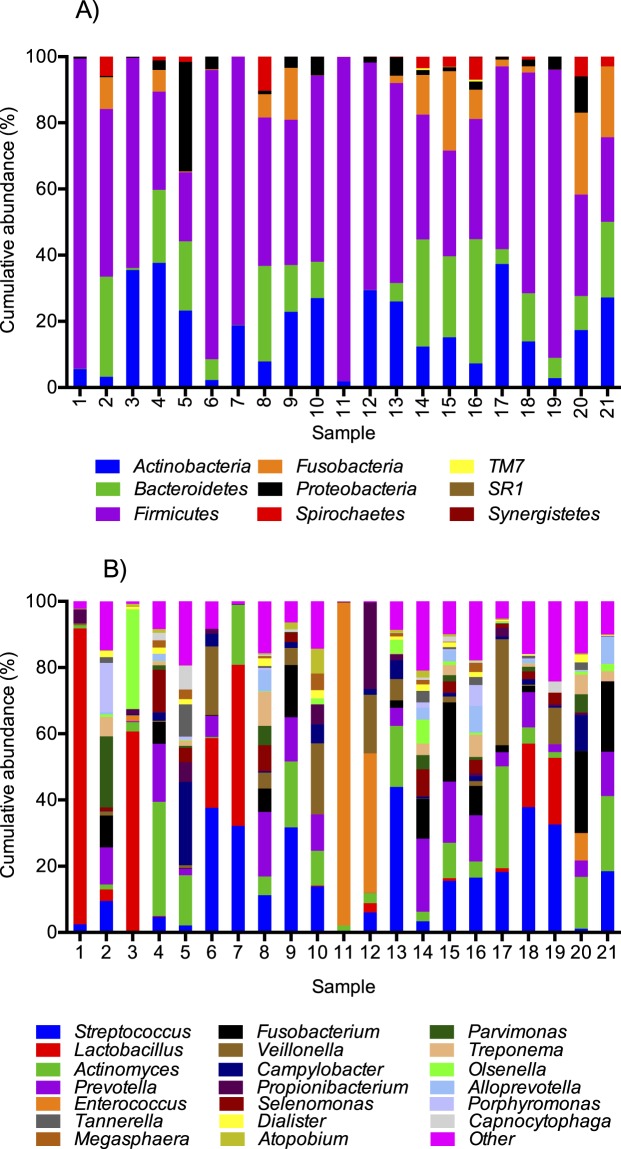
Cumulative abundance of bacterial taxa at the phylum (**A**) and genus (**B**) level for each patient. Only the 20 most abundant genera are shown.

Taxonomic classification of the sequences using the HOMD database showed that nine different phyla were found across all 21 samples with the *Firmicutes* phylum as the most abundant in most samples; average 55% (range: 21–98%) of all the sequences (Fig. 3A and Table S4). Of the nine phyla detected, only *Firmicutes*, *Bacteroidetes* and *Actinobacteria* were detected in all samples. Interestingly, the phylum *Fusobacteria*, which is a normal colonizer of the oral cavity^43^, was absent from two samples and found in low relative numbers (<1%) in eight other samples. Similarly, *Spirochaetes* were also absent from two samples and found in very low proportions (<0.1%) in eight other samples. At a lower taxonomic level, 80 named genera were detected comprising 97% of the total sequences included in the study (Table S4). Furthermore, 26 unnamed genera were found especially in the *Lachnospiraceae* family and in the *Peptostreptococcaceae* family (Table S4). Of the sequences, 0.3% were not assigned to genus level. *Streptococcus*, *Lactobacillus*, *Actinomyces*, *Prevotella*, and *Cutibacterium* (*Propionibacterium*) were the only genera found in all samples. The low number of genera shared is most likely explained by very high proportions of the genera *Lactobacillus* and *Enterococcus* in samples 1 and 11, respectively (Fig. 3B and Table S4). The number of named genera in each sample ranged from 21 genera in sample 6 to 54 in samples 5 and 16 (average 38 genera), but in most of the samples only a few genera were found in relatively high proportions (>1%) (Fig. 3B).

A total of 181 formally described species were detected (corresponding to 75% of all sequences), while 139 undescribed species or taxa classified as an “oral taxon” belonging to a specific named genus in the HOMD were found (corresponding to 14% of all sequences) (Table S4). Most of the undescribed oral taxa belonged to the *Actinomyces* (12), *Prevotella* (15), *Capnocytophaga* (8), *Selenomonas* (12), and *Treponema* (8) genera. Eleven % of the sequences could not be assigned to species level with HOMD.

**Figure 4:**
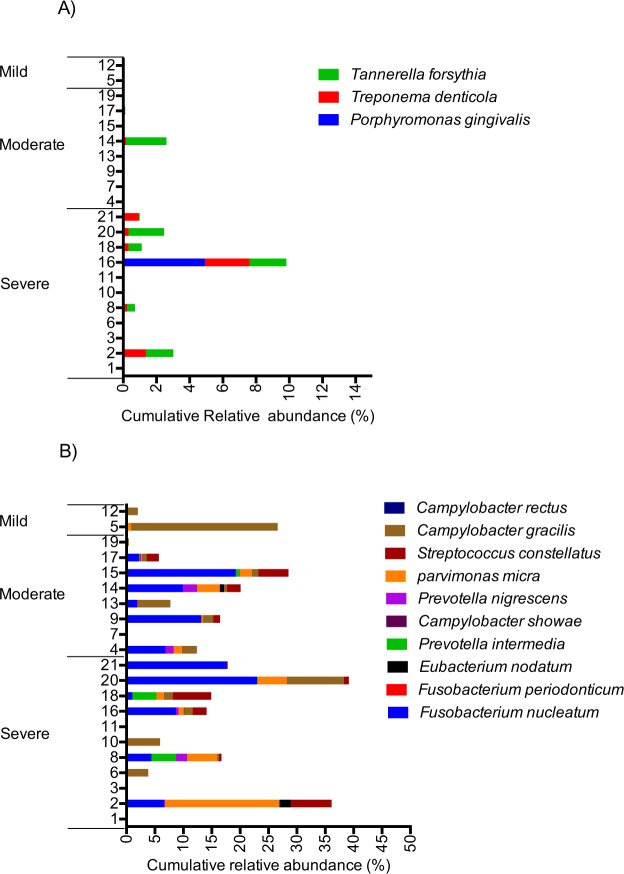
Cumulative abundance of species belonging to the red complex (**A**) and the orange complex (**B**) for each patient. Patients are ordered according to the severity of their periodontitis. The three subspecies of *F*. nucleatum included in the orange complex (subsp. *nucleautm*, subsp. *polymorphum*, subsp. *vincentii*) are grouped.

The number of named species and unnamed oral taxa found in each sample ranged from 33 in sample 1 to 205 in sample 16 (mean = 113). The three most abundant species were *Enterococcus durans*, *Lactobacillus crispatus*, and *Veillonella parvula*. However, only the latter was found in most samples and only *Cutibacterium* (*Propionibacterium*) *acnes* and *Campylobacter gracilis* were found in all samples (Table S4). While *Campylobacter gracilis* was relatively abundant in several samples, *C*. *acnes* was found only in minor proportions in most samples. The three members of the red complex (*Porhyromonas ginigivalis*, *Tannerella forsythia*, *Treponema denticola*), a group of bacteria significantly associated with severe periodontal disease^44^ were found in very low proportions in most samples, with the exception of sample 16 where species from the red complex accounted for nearly 10% of the total community (Fig. 4A). However, if one of the species was found in larger proportions (>1%), at least two of the species belonging to the red complex were always detected. Bacteria belonging to the orange complex^44^ (*Fusobacterium nucleatum* (subspecies *polymorphum*, subspecies *nucleatum*, subspecies *vincentii*), *Fusobacterium periodonticum*, *Prevotella intermedia*, *Prevotella nigrescens*, *Parvimonas micra* (formerly *Peptostreptococcus micros*), *Streptococcus constellatus*, *Eubacterium nodatum*, *Campylobacter showae*, *Campylobacter gracilis*, *Campylobacter rectus*) were more abundant in most samples and accounted, in total, for an average of 12.8% (range: 0.0002–39.2%) of the individual microbial communities (Fig. 4B). Three of the orange complex species (*C*. *rectus*, *C*. *showae*, and *F*. *periodonticum*) were not found in any of the samples. Interestingly, when species belonging to the orange complex accounted for less than 5% of the total population, as in seven of the samples, species belonging to the red complex were completely absent. While there was no association between the presence of the bacteria in the orange complex and the severity of periodontitis of our subjects, the bacteria in the red complex were, with one exception, only found in higher proportions (>1%) in subjects with severe periodontitis (Fig. 4A and B).

### Comparison of all data sets

We compared our data set with data sets from three other studies to investigate whether the subgingival microbial composition of the subjects with periodontitis and cirrhosis was different from that of other patients with periodontitis or healthy controls with no signs of periodontitis. As a consequence of the inclusion of other datasets, we had to reduce our own datasets to just 4000 reads per sample and with an average read length of 198 bp (see methods section for detailed description). The composition of the microbial community at the phylum level is comparable using our original dataset and the reduced dataset indicating that our findings were robust (Fig. S3). However, the average number of OTUs was reduced from 218 in the original dataset to 163 in the dataset rarefied to 4000 reads. The OTU level of our samples was comparable to that of periodontitis cases from all three published studies, while the mean number of OTUs from healthy samples from both of the previous studies was significantly lower (t-test, p < 0.01) than that of our dataset (Fig. S4A). In contrast, the NPShannon diversity index was comparable for our dataset and the datasets from healthy controls of the Abusleme and Griffen studies, while it was significantly lower (p < 0.01) than found for periodontitis patients from all three included studies (Fig. S4B).

**Figure 5:**
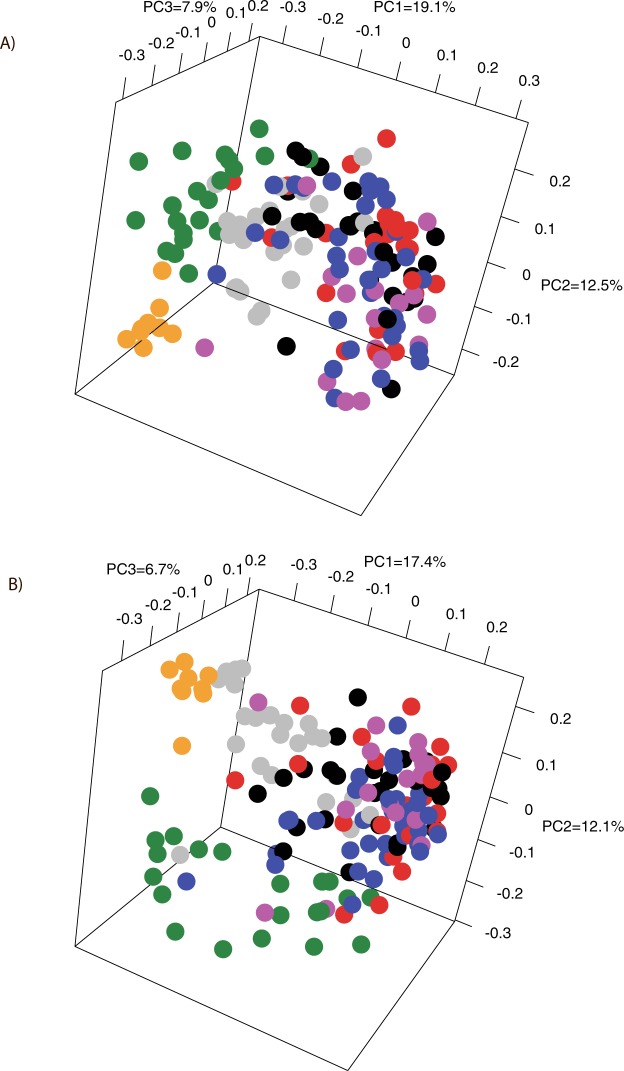
PCoA plots comparing the subgingival plaque bacterial communities from our patients with liver cirrhosis and periodontitis with periodontitis patients and healthy controls from the studies of Hong *et al*.^37^, Griffen *et al*.^35^, and Abusleme *et al*.^36^. The percentage of variation explained by each principal coordinate (PC) is indicated on the axes. Each point represents a microbial community. The plots are based on the OTU structure (ThetaYC calculator) using 98.5% sequence similarity for clustering (**A**) and on Unweighted UniFrac distances from the phylogenetic tree generated from the Clearcut program implemented in the Mothur software (**B**). Samples from our liver cirrhosis patients are shown in green, healthy control samples from the Abusleme study are shown in orange, periodontitis patients from the Abusleme are shown in purple, periodontitis samples from the Hong study are shown blue, periodontitis samples from deep sites from the Griffen study are shown in red, periodontitis samples from shallow sites from the Griffen study are shown in black, and healthy control samples from the Griffen study are shown in grey.

We compared the beta-diversity of the datasets by generating PCoA plots of the unweighted UniFrac distances of the phylogenetic tree generated from the Clearcut program and the ThetaYC distances of the OTUs clustered at a 98.5% similarity level. From Fig. 5A and B it is clearly evident that our samples had a different community structure from that of the other included datasets of periodontitis patients. Similarly, datasets from the healthy controls of the Abusleme study and the Griffen study were clearly distinct from our dataset as well as from the datasets from periodontitis patients (Fig. 5A and B). On the other hand, all four datasets from patients with periodontitis were not clearly separated from each other by both PCoA plots. The separation observed by both PCoA plots was supported by PERMANOVA analysis with p-values below 0.001 between our cirrhosis patients and all the other datasets. PERMANOVA revealed borderline significant differences between the datasets from periodontitis patients with p-values between 0.05–0.06 except between the datasets of deep sites and shallow sites of the Griffen study where p-values from the PERMANOVA analysis were non-significant (p-values between 0.2 and 0.3). Although the PERMANOVA analysis revealed borderline significance between the downloaded periodontitis datasets, they were pooled to detect potential taxonomic differences between our samples and the periodontitis samples. The same was done for the two datasets from healthy controls.

**Figure 6:**
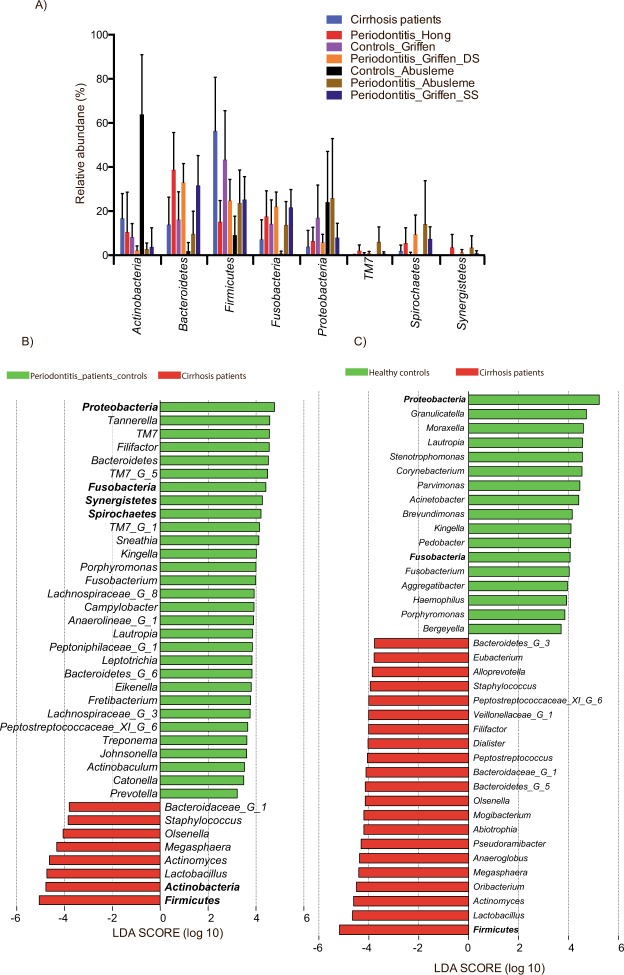
Relative abundance with standard deviation of the most abundant phyla detected in the groups after collectively processing of the reads from the present study and the studies of Hong^37^, Griffen^35^ and Abusleme^36^ (**A**) and differentially abundant bacterial phyla and genera identified by linear discriminant analysis (LDA) coupled with effect size measurements (LEfSe) between our samples and the pooled samples with periodontitis (**B**) and the pooled healthy control samples (**C**) from the three included studies. Only taxa that met the significant linear discriminant analysis threshold of 3.5 are shown. Phyla are written in bold.

Taxonomic classification of the sequences from the compared datasets showed a remarkably different microbial composition between our samples and the three datasets from patient with periodontitis at the phylum level (Fig. 6A). Clearly, *Firmicutes* was more abundant in our samples than in all other datasets, while *Spirochaetes*, *Fusobacteria*, and *Bacteroidetes* were less abundant than in all the published datasets generated from patients with periodontitis. These three phyla were also less abundant in the healthy controls of the Abusleme study and the Griffen study when compared to the patients with periodontitis from the same studies (Fig. 6A). Using linear discriminant analysis (LDA) coupled with effect size measurement (LEfSe) to detect differentially abundant bacterial taxa (phyla, genera, species), the same differences at the phylum level were confirmed in addition to TM7 and *Synergistetes* being more abundant in periodontitis patients (Fig. 6B). Such major differences at the highest taxonomic level clearly indicate that the two groups have a completely different bacterial community structure, which is supported also by the high number of differentially abundant taxa at a lower level between the two groups (Figs 6B and S5A). In contrast, only *Firmicutes* (cirrhosis patients) and *Proteobacteria* (healthy controls) were differentially abundant at the phylum level between cirrhosis patients and healthy controls (Fig. 6C). Interestingly, the species belonging to the red complex, *T*. *forshytia*, *T*. *denticola* and *P*. *gingivalis*, were found to be significantly more abundant in periodontitis patients than in our cirrhosis patients with periodontitis but not in samples from healthy controls (Fig. S5A and B). In addition, the orange-complex species *F*. *nucleatum*, *P*. *intermedia*, *P*. *nigrescens*, and *C*. *rectus* were also more abundant in patients with periodontitis than in the cirrhosis patients, while *S*. *constellatus* (cirrhosis), *F*. *nucleatum subsp*. *polymorhum* (healthy controls) and, *C*. *showae* (healthy controls) were found in different abundancies in cirrhosis patients and healthy controls (Fig. S5A and B). Due to relatively short sequences included in these analyses, comparison at the species can be problematic. However, most of the genera to which the species in the red and orange complex belong (*Treponema*, *Tannerella*, *Porphyromonas*, *Fusobacterium*, *Prevotella*, *Eubacterium*), were also more abundant in periodontitis patients than in cirrhosis patients with periodontitis while *Fusobacterium* and *Porphyromonas* were actually more abundant in healthy controls than in cirrhosis patients (Figs 6B,C and S6).

## Discussion

This is the first report on the subgingival microbiome of liver cirrhosis patients suffering from periodontitis. We used the 2 × 300 bp V3 Miseq Illumina kit for sequencing of the V1-V3 region of the 16S rRNA gene. While the long reads, theoretically, result in a better taxonomic resolution, the method has been criticised for generating sequencing errors leading to too many unique reads and, ultimately, an overestimation of the number of OTUs present in the samples^45^ (see this blog http://blog.Mothur.org/2014/09/11/Why-such-a-large-distance-matrix/ for a discussion of the problem). To deal with these problems, our sequence provider developed an in-house protocol for sequencing of 2 × 300 bp on the HiSeq. 2000 instrument. Together with our rigorous processing of the reads, we consider that the error rate in our final reads used for downstream analysis was reduced to a minimum. This is supported by the number of OTUs and diversity indexes found in our samples, which were in agreement with previous studies of the subgingival plaque microbiome in periodontitis and gingivitis patients using a 98.5% 16S rRNA gene sequence identity to define OTUs^34,46^. Although different sequencing methods theoretically may affect the outcome of microbiome studies, previous studies have shown that 454 pyrosequencing and Illumina sequencing result in only minor insignificant taxonomic differences^47,48^. Therefore, we trust our data and consider our findings to be valid.

Our patients suffered from liver cirrhosis mainly due to present or former alcohol abuse. However, they were very diverse in many other clinical and medical parameters that might affect the composition of the subgingival microbiome. We did not find that the plaque microbiome was affected by a single clinical or medical factor but was more likely affected by several concurrent factors. More than half of our patients had received antibiotics (including rifaximin) within half a year before the sampling, and many of the patients had received antibiotics close to the sampling. Antimicrobial therapy may be the single factor that affects the microbiome most on both a short-term and long-term scale^49^. However, a study by Zaura *et al*.^50^, in which the authors studied the changes in the faecal and salivary microbiome due to antibiotics in 66 healthy adults, revealed a profound and long-term change in the faecal microbiome, while the salivary microbiome was more resilient and not considerably affected. This is in accordance with the absence of a specific alteration of the composition of the microbiome or a reduction in the number of observed OTUs in our patients receiving antibiotics prior to the sampling.

The mean age of patients examined in this study was somewhat higher than in most studies of periodontal disease etiology, but although the oral microbial community changes during the lifetime, the most significant age-related changes take place during childhood and adolescence and only minor changes are seen during adulthood^51^. General dental hygiene affects the composition of the oral microbiome and poor dental hygiene favors a microbial community as associated with periodontitis. However, the dental hygiene observed in our patients was not different from that previously reported for periodontitis patients.

Another factor that might disturb the microbiome in liver cirrhosis patients is lactulose, which is used as a prebiotic for patients with liver cirrhosis and hepatic encephalopathy^52–54^. Lactulose acidifies the colonic pH, which renders the environment hostile to the urease-producing *Klebsiella* and *Proteus* species, while promoting non-urease-producing and aciduric lactobacilli and bifidobacteria^55^. In the few studies that have investigated the direct effect of lactulose on the gut microbiome in liver cirrhosis patients no major changes of the gut microbiome composition were found^56,57^. Interestingly, however, *Lactobacillus* species were very abundant in several of our patients accounting for more than 19% (19–89%) in six of our samples, of which four had received lactulose, suggesting that lactulose may increase the oral abundancy of this genus in patients with liver cirrhosis. *Lactobacillus* has not been found to be abundant in the oral cavity of otherwise healthy periodontitis patients or controls^43,58,59^, but is generally considered to have a beneficial role in periodontal diseases by suppressing the growth of periodontal pathogens by its antimicrobial and anti-inflammatory properties^5,60^. This might explain the low abundances of species from the red and orange complexes in samples rich in *Lactobacillus* species. However, a recent study^61^ found that *Lactobacillus gasseri* was highly abundant in periodontitis patients and especially in progressing periodontitis patients but not in healthy controls. If confirmed, these findings suggest different properties of *Lactobacillus* species or even clones of the same species^62^ and do not exclude that lactobacilli are involved in periodontitis development in specific patients like liver cirrhosis patients when other more common periodontal pathogens are suppressed.

In spite of individual differences, the subgingival microbiome in periodontitis patients is distinct from that of healthy people^43,58,59^. Several studies have found that phyla like *Bacteroidetes*, *Spirochaetes*, and *Fusobacteria* are more abundant in patients with periodontitis^35,36,63^. Interestingly, we did not find high proportions of these phyla in the subgingival microbiota associated with periodontal attachment loss in cirrhotic patients. Our samples were dominated by *Firmicutes* and to a lesser extend *Actinobacteria*, both of which have been associated with health^58,63^. This conclusion is supported by comparison of our data with those of three other studies, which showed that the composition of our samples was more similar to the included healthy control samples at the phylum level. *Synergistetes* and TM7 were almost absent from our samples, and both phyla have been suggested to be associated with periodontitis^64,65^. The absence of these phyla could be a methodological issue, as proposed by Griffen *et al*.^35^, who observed a lower detection rate of these two phyla when using the V1 regions of the 16 S rRNA gene compared to the V4 region for taxonomic assignment. However, we find it more likely that the two phyla were truly absent from our samples in accordance with the conclusion supported by the PCoA analysis that the microbial compositions of our samples are more similar to that found in healthy subjects.

Theoretically, the pocket depth of the sampling sites might explain the differences in the microbial communities between our patients with liver cirrhosis and patients from the compared data sets. While the PD and CAL from all teeth of the patients were comparable between our patients and the comparison studies, the PD at the sampling sites varied between the studies. However, Griffen *et al*.^35^ showed that the abundance of disease-associated bacterial species was almost similar between shallow sites (mean PD 3.2 mm) and deep sites (mean PD 6.2 mm) from the same patients, which was also confirmed by our analysis. Similarly, Hong *et al*.^37^ found that a higher mean overall PD was a better prediction parameter for periodontal disease-associated bacteria than the PD at sampling sites. In conclusion, these findings suggest that overall periodontal status of the patient more accurately reflects the subgingival plaque community than the PD/CAL at the actual sampling site. In conclusion, we acknowledge that both sampling, sequencing, and clinical differences of the included patients from the compared datasets may impact the microbiological findings. However, it is conceivable that such differences are relatively minor and that the observed differences in the bacterial composition between our patients and the included datasets are real and associated with the liver cirrhosis and ensuing immune dysfunction in our patients.

It is well established that liver cirrhosis patients become immunocompromised, which is responsible for many of the complications observed, including bacterial infections^66,67^. The changes in the gut microbiota in liver cirrhosis patients have been proposed to be caused by a combination of the direct effect of the liver on the gut as well as by their immunocompromised status^22,24,55^. Recent studies showed that the oral microbiome associated with periodontitis differs greatly between HIV positive and HIV negative patients^46,68,69^. In the study by Noguera-Julian *et al*.^68^ *Neisseria* species were enriched in HIV-positive patients. Interestingly, genera belonging to the red complex (*Tannerella*, *Treponema*, *Porhyromonas*)^44^ were found in low amounts in both HIV-positive and HIV-negative patients. However, in two other studies by Kistler *et al*.^46^ and Goldberg *et al*.^70^ no overall differences in the oral bacterial community structure was found between well-controlled HIV-positive children and HIV-negative children as well as HIV-positive adults undergoing highly active antiretroviral therapy (HAART) and HIV-negative adults, respectively. These studies clearly demonstrate that the immune status of the liver cirrhosis patients may alter the bacterial community, in accordance with what we observed in the oral cavity.

Complete appreciation of the causal relationship between liver cirrhosis and periodontal disease requires information on the exact time of debut of the two diseases. As both diseases are characterized by slow progression and may be influencing the immune system long before a clinical diagnosis is made, it would be almost impossible to determine the order of the two diseases. On the other hand, it has been shown that periodontal diseases are more prevalent in patients with liver cirrhosis patients than in similar patient groups without liver cirrhosis^71^, which suggests a causal relationship between liver cirrhosis and periodontal diseases.

Periodontal disease is a result of dysbiosis, which in most patients is caused by an increased microbial challenge due to various factors associated with modern life-style^5,72^. However, dysbiosis may also be the result of reduced host resistance to the microbial challenge. It is conceivable that the observed periodontal microbiota in cirrhotic patients, which is compatible with periodontal heath in healthy individuals, is capable of causing inflammation and attachment loss when affected only by a compromised immune defense.

An increasing body of literature links the oral microbiome with diseases in other parts of the human body^10^. Studies of the gut microbiome in patients with liver cirrhosis show an increased abundance of some genera of oral origin like *Veillonella*, *Prevotella*, *Neisseria*, and *Haemophilus*, and suggest that salivary dysbiosis in these patients may be linked to an oral pro-inflammatory environment, systemic inflammation, and constitute a risk for subsequent liver-related hospitalization^24,27^. Interestingly, Aberg and Helenius-Hietala^73^ proposed that periodontitis may be of special interest in liver cirrhosis patients for the translocation of oral bacteria to the gut and for the risk of subsequent liver-related hospitalization. This may be due to the release of periodontal bacteria and their toxins into the circulation during periodontitis^14^. Reports have shown that dental diseases accelerated the progression of liver disease and the overall mortality in liver cirrhosis patients^74,75^.

In this study, we found that that the subgingival plaque microbiome in patients with liver cirrhosis and periodontitis differed from that of otherwise healthy patients with periodontitis. Especially, bacterial species normally associated with periodontitis from the red complex and to a lesser extend from the orange complex were less abundant in our samples. We hypothesize that periodontitis in patients with liver cirrhosis is associated with bacteria normally compatible with health due to the dysbiotic situation caused by the immunocompromised status of most liver cirrhosis patients. Our findings emphasize that dysbiosis leading to periodontal disease may be due either to changes in the composition of the microbiota or to changes of the host’s immune responses or to both. Our study strengthens the connection between liver cirrhosis and oral health. Liver cirrhosis patients often develop oral diseases like periodontitis and oral diseases in liver cirrhosis patients may aggravate their prognosis. Therefore, it is vital to follow the oral health of liver cirrhosis patients, and further studies have to be conducted to establish the role of the oral microbiome in the pathogenesis of liver cirrhosis.

## Electronic supplementary material

Supplementary figures and tables

Table S2

Table S3

Table S4
