# Supplementary material for: Unique subgingival microbiota associated with periodontitis in cirrhosis patients
Source: Sci Rep. 2018 Jul 16;8:10718. doi: 10.1038/s41598-018-28905-w (PMC6048062; doi:10.1038/s41598-018-28905-w)
Supplement: Supplementary file 1 — Supplementary figures and tables [file 41598_2018_28905_MOESM1_ESM.pdf]

Unique subgingival microbiota associated with periodontitis in cirrhosis patients

Anders Jensen[1*]

Lea Ladegaard Grønkjær[2]

Palle Holmstrup[3]

Hendrik Vilstrup[2]

Mogens Kilian[1]

1. Department of Biomedicine, Faculty of Health, Aarhus University, Aarhus, Denmark.

2. Department of Hepatology and Gastroenterology, Aarhus University Hospital, Aarhus, Denmark.

3. Section of Periodontology, Microbiology, and Community Dentistry, Department of Odontology, Faculty of Health and Medical Sciences, University of Copenhagen, Copenhagen, Denmark.

* corresponding author: contact information Aarhus University Bartholins Allé 6 8000 Aarhus C Denmark, +4526851045, aj@biomed.au.dk

Supplementary information (Supplementary Figures S1-S6; Supplementary Table S1)

Figure legends

Supplementary Figure S1. Rarefaction curves of the samples from the included datasets. Only values up to 9,000 sequences are shown. Samples were rarefied to 4,000 sequences were chosen as a trade-off between keeping most of the samples and keeping most of the diversity. Griffen_DS: Deep sites, Griffen_SS: Shallow sites.

Supplementary Figure S2. PCoA plots the subgingival plaque bacterial communities from each of our patients with liver cirrhosis and periodontitis. The percentage of variation explained by each principal coordinate (PC) is indicated on the axes and colored according to etiology (**A**), severity of

periodontitis (**B**), gender (**C**), daily alcohol intake (**D**), antibiotics within the last ½ year (**E**), and lactulose use (**F**). Each point represents a microbial community. The plots are based on the OTU structure (thetaYC calculator) using 98.5% sequence similarity for clustering.

Supplementary Figure S3. Comparison of the original dataset and the reduced dataset after reduction of the number of reads in each sample to 4000.

Supplementary Figure S4. Microbial richness and diversity based on the no. of observed OTUs (**A**) and the NPShannon diversity index (**B**) in each of the compared groups. Students t-test are used for identifying differences between the groups (**<0.01; ***<0.001). Griffen_DS: Deep sites, Griffen_SS: Shallow sites.

Supplementary Figure S5. Differentially abundant bacterial phyla and genera identified by linear discriminant analysis (LDA) coupled with effect size measurements (LEfSe) between our samples and the pooled samples with periodontitis from the three comparison studies[34,35,36] (**A**) and the pooled healthy control samples (**B**) from the Griffen study[34] and the Abusleme study[35]. Only taxa that met the significant linear discriminant analysis threshold of 3.5 are shown.

Supplementary Figure S6. Relative abundance of most abundant phyla detected in the groups after collectively processing of the reads from the present study and the studies of Hong study[36], Griffen study[34] and Abusleme study[35] . Bars represent standard derivation values for each bar. Griffen_DS: Deep sites, Griffen_SS: Shallow sites.

Additional Files (XLSX-files)

Supplementary Table S2: Clinical and sequence characteristics of all included samples from the present study and the three comparison studies

Supplementary Table S3: Taxonomic classification of OTUs, the relative abundance and a representative sequence for each OTU

Supplementary Table S4: Unedited output from Mothur of the taxonomic classfication of the sequences in each sample and the relative abundance of phyla, genera and species in the 21 cirrhosis patient samples

Figure S1

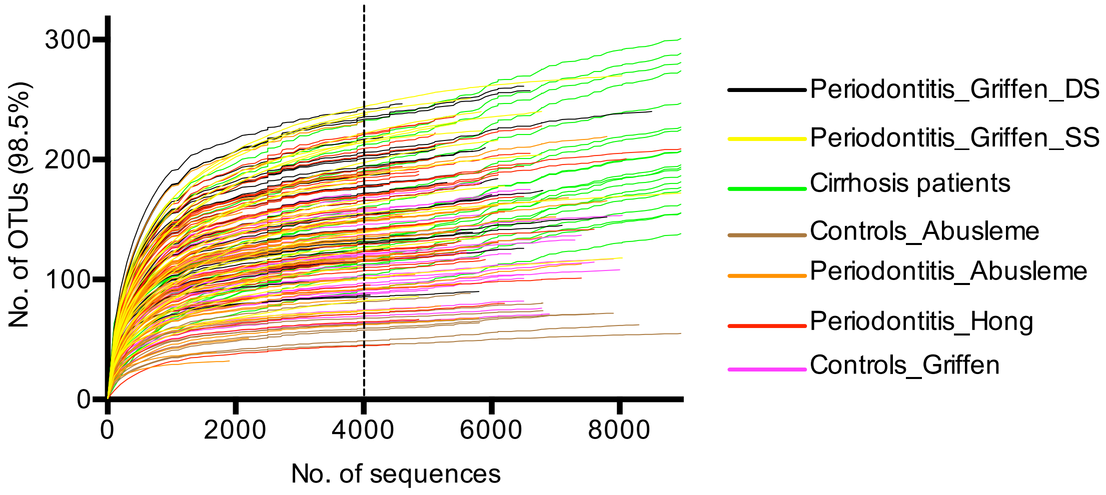

No. of OTUs (98.5%)
300
200
100
0
0
2000
4000
6000
8000
No. of sequences
Periodontitis_Griffen_DS
Periodontitis_Griffen_SS
Cirrhosis patients
Controls_Abusleme
Periodontitis_Abusleme
Periodontitis_Hong
Controls_Griffen

Figure S2

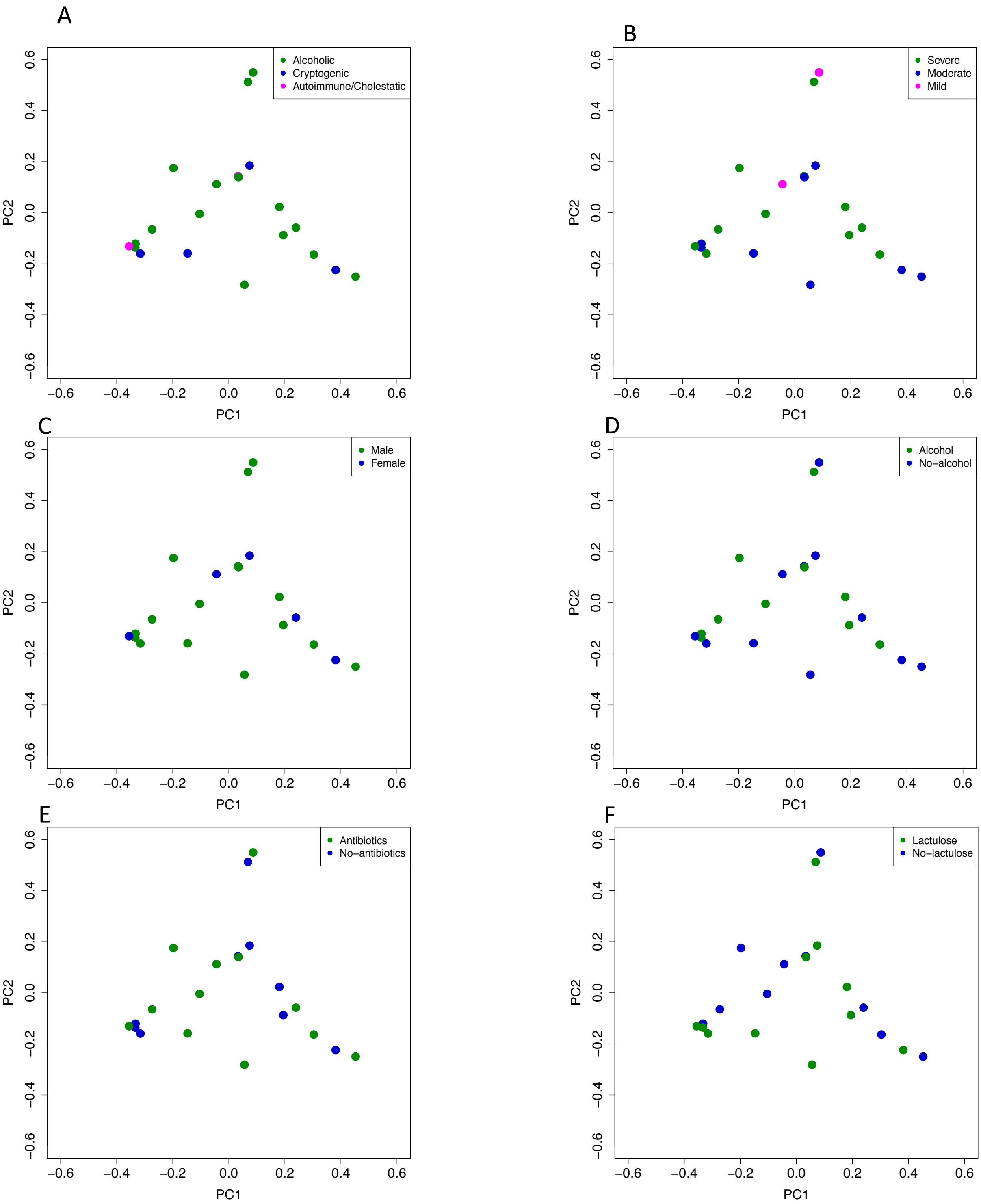
A
Alcoholic
Cryptogenic
Autoimmune/Cholestatic
B
Severe
Moderate
Mild
C
Male
Female
D
Alcohol
No−alcohol
E
Antibiotics
No−antibiotics
F
Lactulose
No−lactulose
PC1
PC2
−0.6
−0.4
−0.2
0.0
0.2
0.4
0.6

Figure S3

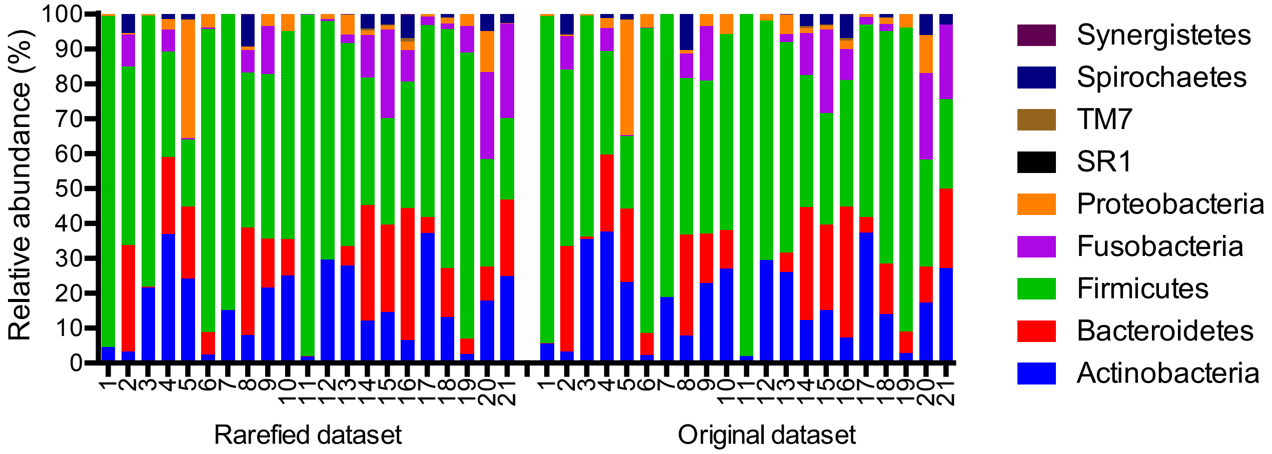
Relative abundance (%)
100
90
80
70
60
50
40
30
20
10
0
1 2 3 4 5 6 7 8 9 10 11 12 13 14 15 16 17 18 19 20 21
Rarefied dataset
1 2 3 4 5 6 7 8 9 10 11 12 13 14 15 16 17 18 19 20 21
Original dataset
Synergistetes
Spirochaetes
TM7
SR1
Proteobacteria
Fusobacteria
Firmicutes
Bacteroidetes
Actinobacteria

Figure S4

A

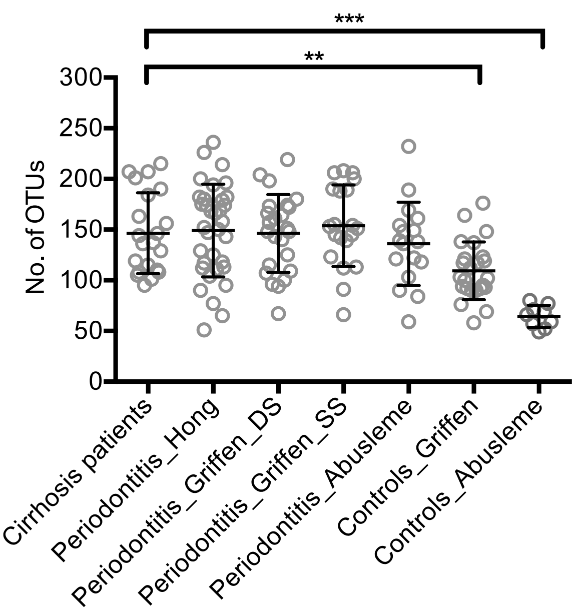

***
**
No. of OTUs
300
250
200
150
100
50
0
Cirrhosis patients
Periodontitis_Hong
Periodontitis_Griffen_DS
Periodontitis_Griffen_SS
Periodontitis_Abusleme
Controls_Griffen
Controls_Abusleme

B

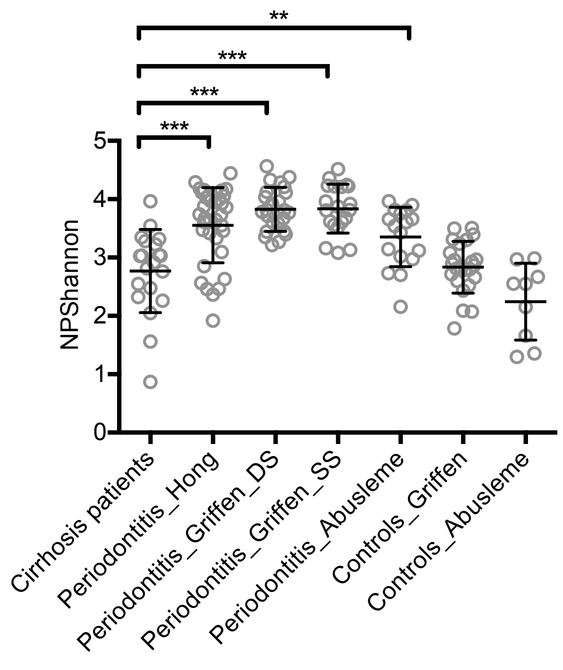

**
***
***
***
NPShannon
5
4
3
2
1
0
Cirrhosis patients
Periodontitis_Hong
Periodontitis_Griffen_DS
Periodontitis_Griffen_SS
Periodontitis_Abusleme
Controls_Griffen
Controls_Abusleme

Figure S5

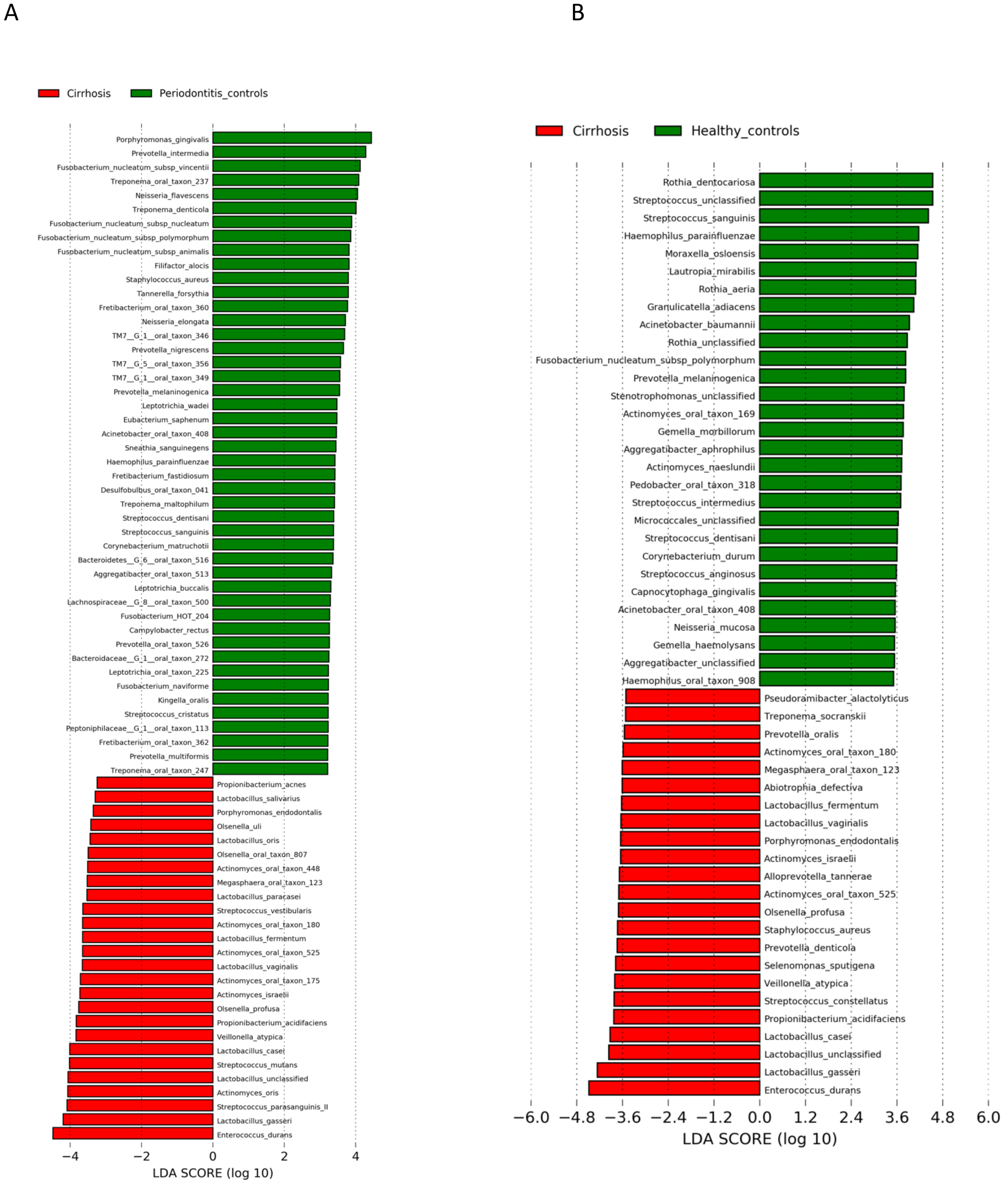

A
Cirrhosis
Periodontitis_controls
Porphyromonas_gingivalis
Prevotella_intermedia
Fusobacterium_nucleatum_subsp_vincentii
Treponema_oral_taxon_237
Neisseria_flavescens
Treponema_denticola
Fusobacterium_nucleatum_subsp_nucleatum
Fusobacterium_nucleatum_subsp_polymorphum
Fusobacterium_nucleatum_subsp_animalis
Filifactor_alocis
Staphylococcus_aureus
Tannerella_forsythia
Fretibacterium_oral_taxon_360
Neisseria_elongata
TM7__G_1__oral_taxon_346
Prevotella_nigrescens
TM7__G_5__oral_taxon_356
TM7__G_1__oral_taxon_349
Prevotella_melaninogenica
Leptotrichia_wadei
Eubacterium_saphenum
Acinetobacter_oral_taxon_408
Sneathia_sanguinegens
Haemophilus_parainfluenzae
Fretibacterium_fastidiosum
Desulfobulbus_oral_taxon_041
Treponema_maltophilum
Streptococcus_dentisani
Streptococcus_sanguinis
Corynebacterium_matruchotii
Bacteroidetes__G_6__oral_taxon_516
Aggregatibacter_oral_taxon_513
Leptotrichia_buccalis
Lachnospiraceae__G_8__oral_taxon_500
Fusobacterium_HOT_204
Campylobacter_rectus
Prevotella_oral_taxon_526
Bacteroidaceae__G_1__oral_taxon_272
Leptotrichia_oral_taxon_225
Fusobacterium_naviforme
Kingella_oralis
Streptococcus_cristatus
Peptoniphilaceae__G_1__oral_taxon_113
Fretibacterium_oral_taxon_362
Prevotella_multiformis
Treponema_oral_taxon_247
Propionibacterium_acnes
Lactobacillus_salivarius
Porphyromonas_endodontalis
Olsenella_uli
Lactobacillus_oris
Olsenella_oral_taxon_807
Actinomyces_oral_taxon_448
Megasphaera_oral_taxon_123
Lactobacillus_paracasei
Streptococcus_vestibularis
Actinomyces_oral_taxon_180
Lactobacillus_fermentum
Actinomyces_oral_taxon_525
Lactobacillus_vaginalis
Actinomyces_oral_taxon_175
Actinomyces_israelii
Olsenella_profusa
Propionibacterium_acidifaciens
Veillonella_atypica
Lactobacillus_casei
Streptococcus_mutans
Lactobacillus_unclassified
Actinomyces_oris
Streptococcus_parasanguinis_II
Lactobacillus_gasseri
Enterococcus_durans
−4
−2
0
2
4
LDA SCORE (log 10)
B
Cirrhosis
Healthy_controls
Rothia_dentocariosa
Streptococcus_unclassified
Streptococcus_sanguinis
Haemophilus_parainfluenzae
Moraxella_osloensis
Lautropia_mirabilis
Rothia_aeria
Granulicatella_adiacens
Acinetobacter_baumannii
Rothia_unclassified
Fusobacterium_nucleatum_subsp_polymorphum
Prevotella_melaninogenica
Stenotrophomonas_unclassified
Actinomyces_oral_taxon_169
Gemella_morbillorum
Aggregatibacter_aphrophilus
Actinomyces_naeslundii
Pedobacter_oral_taxon_318
Streptococcus_intermedius
Micrococcales_unclassified
Streptococcus_dentisani
Corynebacterium_durum
Streptococcus_anginosus
Capnocytophaga_gingivalis
Acinetobacter_oral_taxon_408
Neisseria_mucosa
Gemella_haemolysans
Aggregatibacter_unclassified
Haemophilus_oral_taxon_908
Pseudoramibacter_alactolyticus
Treponema_socranskii
Prevotella_oralis
Actinomyces_oral_taxon_180
Megasphaera_oral_taxon_123
Abiotrophia_defectiva
Lactobacillus_fermentum
Lactobacillus_vaginalis
Porphyromonas_endodontalis
Actinomyces_israelii
Alloprevotella_tannerae
Actinomyces_oral_taxon_525
Olsenella_profusa
Staphylococcus_aureus
Prevotella_denticola
Selenomonas_sputigena
Veillonella_atypica
Streptococcus_constellatus
Propionibacterium_acidifaciens
Lactobacillus_casei
Lactobacillus_unclassified
Lactobacillus_gasseri
Enterococcus_durans
−6.0
−4.8
−3.6
−2.4
−1.2
0.0
1.2
2.4
3.6
4.8
6.0
LDA SCORE (log 10)

Figure S6

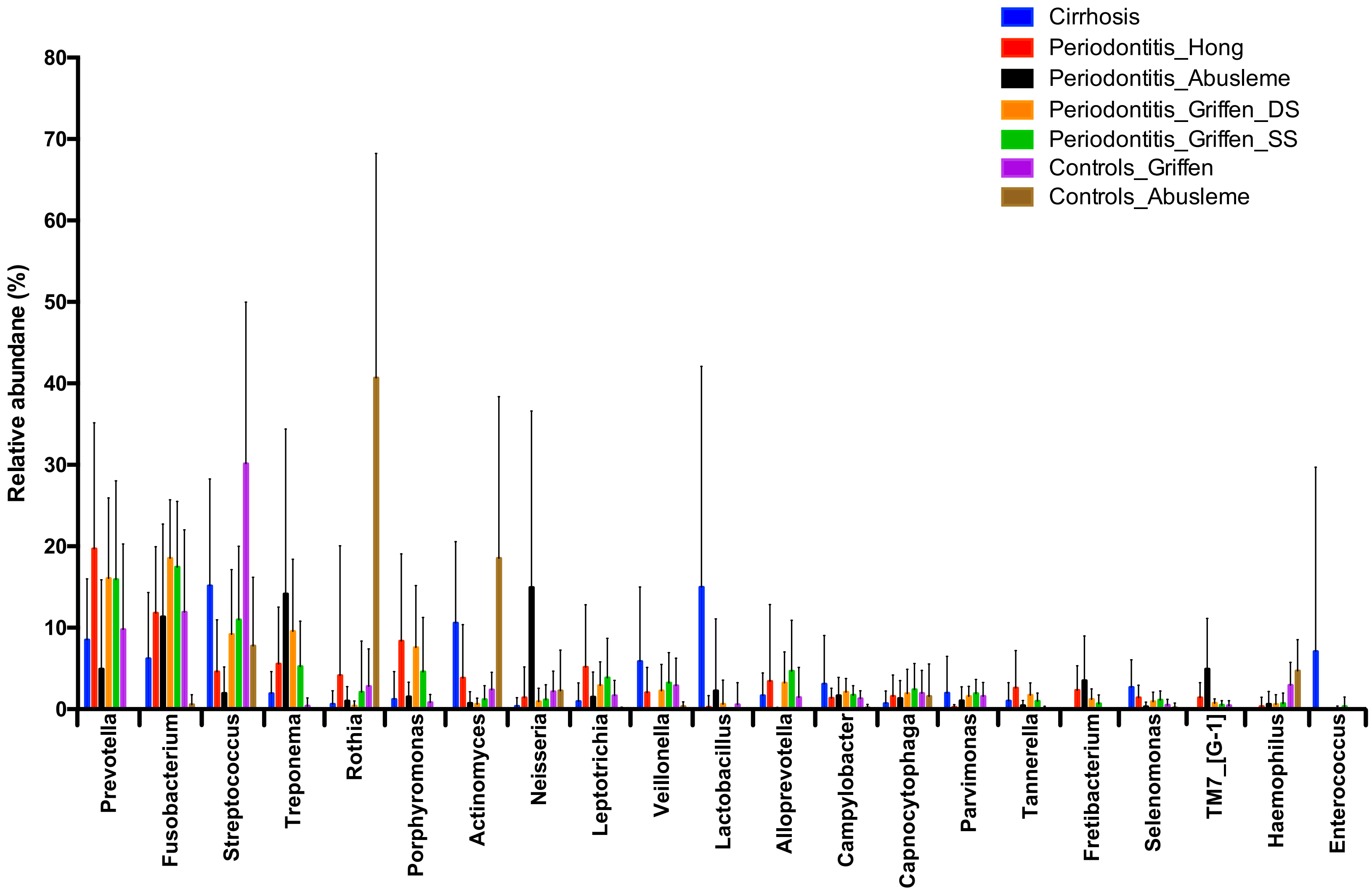
Cirrhosis
Periodontitis_Hong
Periodontitis_Abusleme
Periodontitis_Griffen_DS
Periodontitis_Griffen_SS
Controls_Griffen
Controls_Abusleme
Relative abundane (%)
0
10
20
30
40
50
60
70
80
Prevotella
Fusobacterium
Streptococcus
Treponema
Rothia
Porphyromonas
Actinomyces
Neisseria
Leptotrichia
Veillonella
Lactobacillus
Alloprevotella
Campylobacter
Capnocytophaga
Parvimonas
Tannerella
Fretibacterium
Selenomonas
TM7_[G-1]
Haemophilus
Enterococcus

Table S1. Clinical characteristics of the enrolled patients.

Patients No.	Age	Gender	Aetiology	MELD score	Child-Pugh score	Smoker	Daily alcohol use	Charlson comorbidity index	Lactulose	Pantoprazole	Rifaximin	Antibiotics within the last ½ year	Number of teeth	Probing depth, all teeth (mean, mm)	Clinical attachment level, all teeth (mean, mm)	Bleeding on probing, all sites (%)	Periodontitis
1	75	Female	Alcoholic	7	7	No	No	0	Yes	No	Yes	Yes	24	3,81	4,19	99	Severe
2	84	Male	Alcoholic	8	7	Former	Yes	1	Yes	No	Yes	Yes	21	3,47	4,71	70	Severe
3	77	Male	Alcoholic	14	7	Former	Yes	0	No	No	No	No	24	3,11	3,43	100	Severe
4	62	Male	Cryptogenic	27	8	Former	No	1	No	Yes	Yes	Yes	24	2,87	3,23	50	Moderate
5	68	Female	Alcoholic	10	11	Current	No	0	Yes	No	Yes	Yes	27	2,14	2,31	0	Mild
6	65	Male	Alcoholic	20	12	Former	Yes	1	Yes	No	No	Yes	24	3,05	3,24	100	Severe
7	56	Male	Alcoholic	11	9	Current	No	0	Yes	Yes	No	Yes	6	2,78	2,83	19	Moderate
8	64	Male	Alcoholic	12	10	Former	Yes	1	Yes	Yes	No	Yes	25	3,82	3,83	65	Severe
9	58	Male	Alcoholic	20	12	No	No	0	No	No	No	Yes	28	4,00	4,00	0	Moderate
10	62	Male	Autoimmune /Cholestatic	15	9	Current	No	2	Yes	Yes	No	No	28	2,92	3,36	19	Severe
11	64	Male	Alcoholic	37	10	Current	Yes	0	No	No	No	No	16	4,00	4,50	100	Severe
12	73	Male	Alcoholic	13	6	Former	No	1	Yes	Yes	No	Yes	5	1,70	1,80	7	Mild
13	61	Male	Alcoholic	8	8	Current	Yes	0	No	No	No	Yes	25	1,92	1,95	0	Moderate
14	56	Male	Alcoholic	17	12	Current	Yes	0	Yes	No	Yes	No	29	2,31	2,67	100	Moderate
15	57	Male	Alcoholic	24	9	No	Yes	3	No	No	No	No	27	2,72	2,84	14	Moderate
16	59	Female	Autoimmune /Cholestatic	6	6	Current	No	0	No	Yes	No	Yes	5	4,08	4,29	17	Severe
17	75	Female	Cryptogenic	8	6	Current	No	1	No	No	No	No	6	3,36	3,75	83	Moderate
18	60	Male	Alcoholic	24	11	Current	Yes	0	No	No	No	No	27	3,90	4,25	85	Severe
19	87	Female	Cryptogenic	15	9	No	No	0	No	No	No	No	6	2,29	2,40	5	Moderate
20	59	Male	Alcoholic	6	11	Current	Yes	0	Yes	Yes	No	Yes	26	3,85	4,54	92	Severe
21	62	Male	Cryptogenic	9	7	Former	No	1	No	No	No	No	16	4,05	4,51	24	Severe
